# Supplementary material for: Are carbon emissions trading and green financial instruments synergistic? -Comprehensive quantitative research based on content analysis
Source: PLoS One. 2024 Mar 7;19(3):e0298601. doi: 10.1371/journal.pone.0298601 (PMC10919601; doi:10.1371/journal.pone.0298601)
Supplement: S1 Data — (ZIP) [file pone.0298601.s001.zip › Data/地级市Z_F工作报告.zip!/║┌┴·╜¡/╦τ╗»╩╨/└┤╫╘╣1⁄2╓┌║┼í╛┬φ┐╦╩2╛▌═°í┐_2003.docx]

各位代表:

我代表市政府向大会作工作报告,请予审议。

一、2002年政府工作的简要回顾

过去的一年,面对特殊的形势、特殊的困难和特殊的压力,我们认真贯彻“三个代表”重要思想,按照市委确定的总体思路和部署,自觉接受人大、政协的依法监督和民主监督,紧紧依靠全市人民,解放思想,奋发进取,较好地完成了市一届人大三次会议所确定的各项工作任务目标。

经济建设步伐加快,总体实力不断壮大。全市国内生产总值实现320.2亿元,同比增长7.8%;固定资产投资完成30.2亿元,同比增长3.2%;全口径财政收入完成17.16亿元,一般预算收入实现9.6亿元,同比分别增长4.9%和0.4%;城镇人均可支配收入实现3792.5元,农民人均纯收入达到2024.3元,同比分别增长3.9%和3.5%。工业经济运行质量提高,规模以上工业完成增加值22亿元,实现利税5.5亿元,同比分别增长7.5%和17.4%。农村经济持续发展,农业增加值实现101.1亿元,增长6.5%;粮豆薯总产达到104.5亿斤,增长11.8%;绿色食品总产量、商品量、获得标志认证的产品数量,均居全省前列;畜牧业增加值实现30.2亿元,占农业增加值的比重达到29.9%;乡镇企业实现增加值16.4亿元、税金1.3亿元,同比分别增长8%和6.2%;新增造林面积53万亩,森林覆被率达到13.2%。第三产业有新的突破,完成增加值138.9亿元,增长9.6%;社会消费品零售总额实现102.1亿元,增长4.9%;个体私营经济实现税金3.2亿元,同比增长12%。

改革开放深入推进,发展活力明显增强。全市国有工业企业改制面已达98.7%;盘活资产16亿多元。农村税费改革扎实推进,全口径农民负担由改革前的11.95亿元减到7.63亿元,降低36.1%。撤乡、并村、并校等配套改革也取得明显成效,市、县、乡机构改革圆满结束,各级政府的行政审批制度改革以及财政、粮食流通体制和社会保障制度改革都在积极稳妥地进行。全方位多层次对外开放的格局正在形成,招商引资引智工作有了新进展。全年共引进各类项目586项,其中新上工业项目投产达产47项;引进各类人才366名;外贸进出口总额实现3608万美元,利用外资4210万美元,同比分别增长16.5%和5%。

社会发展成效显著,各项事业全面进步。全市城乡基础设施建设取得新的成效,投资累计完成3.85亿元,同比增长30%。交通、通信、电力建设水平有新的提高。科教兴市战略得到深入实施,科技对经济增长的贡献率达到38.5%,教育改革和发展迈出新的步伐,文化、卫生、体育、广播电视和信息产业有新的发展,国土资源、计划生育、环境保护、民族宗教、外事侨务、残疾人以及民兵预备役和双拥等各项工作也取得了新成绩。

精神文明和民主法制建设得到加强,社会稳定和政治安定的良好局面进一步形成。坚持用先进理论武装教育全体公民,通过广泛开展“新绥化形象年”活动,深入实施《公民道德建设实施纲要》,全面提高了公民道德素质。综合治访、依法治访工作得到强化,到省集体访的批次、人次分别下降65%和43%。深入开展“四五”普法活动,扩大政务、厂务、村务公开范围,提高了基层民主政治建设水平。农村劳动力资源开发取得突破性进展,城镇下岗职工再就业率达到67%。社会保障体系进一步完善,职工养老保险参险率达到99.8%,离退休人员基本养老金发放率达到93%,下岗进入再就业服务中心职工的基本生活费发放率达到100%。社会治安综合治理工作不断加强,安全生产工作杜绝了重特大事故的发生。

市本级发展取得新的成效,中心城市功能有所加强。工业企业完成增加值2.15亿元,实现利税1.05亿元,同比分别增长6%和51.7%。省级工业开发区建设有了新的进展,已经入区建设的三精制药、银娃豆奶等重点项目投产在即。财政总收入实现19908万元,其中一般预算收入完成6868万元,同比分别增长11.2%和17.3%。共引进各类项目42项,引进资金4.6亿元,实际到位资金2.9亿元。市政府还成立专门组织,采取综合措施,加强了市区烟尘污染治理工作,并取得了阶段性成果。东风路改建、中兴东路商业街、一中教学楼、中心血站、中央商城、客运枢纽、妇幼保健院、正大购物中心、小商品批发等一批重点建设项目已竣工投付使用,西二路、红兴水库等大型基础设施建设项目也完成了年度施工任务。

上述成绩的取得,是省委、省政府和市委正确领导、亲切关怀的结果,是全市各级干部和广大人民群众团结奋斗、努力拼搏的成果,是人大代表、政协委员、民主党派、无党派人士、工商联、群众团体等社会各界人士全力支持、热情关心和有效监督的结果,也是驻绥中省直单位、解放军和武警官兵积极参与、鼎力相助的结果。在此,我代表市政府,向所有关心、支持、参与绥化建设的同志们、朋友们,致以崇高的敬意和衷心的感谢!

各位代表,回顾过去一年的工作,我市经济和社会发展取得了很大成绩,但仍然存在不少突出的问题。主要是经济发展的结构性矛盾还相当突出,工业化进程缓慢,工业经济规模小、总量少、竞争力差,对市域经济发展的牵动作用还没有真正形成;经济增长方式还没有得到有效的转变,粗放经营、效益低下的问题还相当普遍,一些地方的财政还相当拮据,难以满足和支撑各项事业的发展及社会保障政策的落实;经济发展环境还不够宽松,行业不正之风、部门利益至上乃至一些消极腐败现象还影响阻碍着经济发展;市本级经济发展缓慢,中心城市的整体功能尚未形成,辐射带动作用难以发挥。特别是我们一些同志的思想解放程度和工作作风还不能完全适应加快发展的需要,运用市场经济规律指导经济工作的意识和能力还不够强,工作落实不到位的现象还比较突出,等等。所有这些问题,都必须在今后的工作中认真加以解决。

二、2003年政府工作的主要任务

今年是全面贯彻党的十六大精神的第一年,也是落实省九次党代会和十届人大一次会议精神,确保完成“十五”计划的关键一年。按照市委一届十一次全委(扩大)会议确定的总体思路和开展“规模推进年”的工作部署,今年政府工作的总体要求是:以邓小平理论和“三个代表”的重要思想为指导,以“六个规模推进”为重点,解放思想,开拓创新,攻坚克难,求真务实,加速结构调整,扩大改革开放,推动科技进步,改善发展环境,转变增长方式,把全市经济社会发展提高到一个新的水平,为全面建设小康社会奠定坚实基础。按照这一总体要求,2003年全市经济和社会发展主要预期目标是:国内生产总值实现349亿元,增长9%,其中一、二、三产业增加值分别增长5.2%、12.2%和9.8%;全口径财政收入实现17.7亿元,一般预算收入实现9.6亿元,按可比口径计算,同比分别增长4.38%和3.4%;全社会固定资产投资增长10%以上;社会消费品零售总额达到109亿元,比上年增长7%;城镇居民人均可支配收入实现4058元,农村人均收入实现2125元,同比分别增长7%和5%。为实现上述目标,要着力抓好以下十个方面工作:

(一)走新型工业化道路,不断壮大工业经济的整体实力

按照党的十六大精神和市委全会的要求,以实施工业经济规模推进为突破口,立足现有基础,挖掘潜在优势,内涵外延并举,合力攻坚,奋力拼搏,加快全市的工业化进程,确保全市工业经济总量能有一个较大突破。

实施项目牵动战略,在壮大工业经济整体规模上实现新突破。依据国家产业政策,牢牢把握市场导向,充分发挥本地优势,积极争上一批国家和省重点扶持的北药开发、绿色环保和资源综合利用项目,大力发展一批具有绥化优势和发展潜力的农副产品精深加工项目、高回报率的财源建设项目和劳动密集型产业项目。在继续抓好3500吨L—乳酸等20个续建项目,促其尽快投产达效的同时,重点推进小牛血清蛋白提取物注射液等35个新建项目,加速构筑“新老并举,多业支撑,特色突出,优势明显”的工业经济新格局,不断壮大全市工业经济的整体规模。

实施联大靠强战略,在培育壮大骨干企业群体上实现新突破。通过采取政策扶大、改革促大、联合造大、引资壮大等多种方式,打造一批具有较大牵动和辐射能力的大公司、大企业、大集团。积极引导青冈龙凤、海伦亚泰、北林继嘉、肇东伊利等企业组建集团。推动鼓励东安化工、海伦啤酒、绿洲精洁米、庆钢集团、北林赛利、参美制药、澳利达制药等已经形成集团或具备较大规模的企业,加速实现低成本扩张,成为产值超亿元、利税超千万的大型企业。重点抓好绥化啤酒与哈啤、参美集团与北京同仁堂、绥棱化工与韩国大宇集团、庆安三松与美国白福润集团、肇东生物制品与山东九环集团等50多对企业的挂靠联合与协作,促进生产要素的合理流动与优化配置。积极培育庆东集团等有潜力、有实力的股份制企业上市经营,拓宽融资渠道,增强发展活力。同时,要克服困难,抓紧完成现有制药企业的GMP认证工作。

实施品牌开发战略,在提高企业竞争力上实现新突破。增强企业的品牌意识、自主开发和创新能力,提高企业管理水平,加大市场深度开发和营销力度,加速培育一批在市场上具有影响力和竞争力的名牌产品,争取再有一二个产品获得国家驰名商标,有一批产品获得省著名商标,以此来扩大市场份额,提高企业核心竞争力,打造更多的优势企业。

实施体制创新战略,在增强企业发展活力上实现新突破。坚持民营化、股份化、集团化的方向,加快“国退民进”的步伐。对已经转制的企业按照现代企业制度进行全方位的规范,进一步优化企业法人治理结构,确保改制后的企业焕发出应有的活力;采取股、破、租、包等各种手段,加大危困企业的改组力度,实现破产重组、售后重组。强化现代企业管理运行机制,实现体制创新,不断提高企业的经营力、创新力和竞争力。同时,按照中央和省的统一部署,坚持责、权、利相统一和管资产、管人、管事相结合的原则,改革和完善国有资产管理体制,促进资产整合,增强资本运营能力。

(二)千方百计增加农民收入,全面提高农业和农村经济的竞争力

按照党的十六大关于建设现代化农业、发展农村经济和增加农民收入的重大部署,以及中央关于新阶段农业和农村工作的战略决策,深入实施推进全市农村改革和发展的各项重大措施,确保农业和农村经济有一个新的发展和跨越。

优化布局,突出重点,深入推进农业和农村经济结构的战略性调整。按照国家建设优势农产品产业带,加快农业区域布局调整的总体要求,推进优势农产品和特色农产品向优势产区集中,构建具有较强竞争力的各具特色的产业带。积极引导农民以市场为导向,扩大优质、高效、生态、安全农产品的生产规模,进一步推进绿色食品产业升级,努力扩大基地规模,提高精深加工能力,搞好品牌整合,提高我市绿色食品的知名度和市场占有率。今年,全市经国家认证的A级绿色食品面积争取达到150万亩,按照A级标准组织生产面积达到400万亩,其中AA级绿色食品监测面积达到20万亩。抓住国家实施“大豆振兴计划”的机遇,积极把大豆产业做强。进一步提升畜牧业在农村经济中的地位,在调结构、抓大户、上龙头、搞服务上下功夫,努力实现优质、高效、生态、安全的目标,加快畜牧经济总量扩张。围绕生态农业建设,有计划地搞好退耕还林还草,大力发展林草经济,使之成为农村经济发展的重要产业。宽领域、多形式地推动农村剩余劳动力向二、三产业转移,搞好埠外劳务输出,最大限度地转移富余劳动力,拓宽农民增收渠道。

抓住关键,强化龙头,深入推进农业产业化经营。集中力量重点扶强一批、发展一批、改造提升一批、嫁接整合一批农副产品加工龙头企业,延伸产业链条。注重企业与基地的有效对接,突出搞好水稻、大豆、玉米、亚麻、马铃薯和奶牛、生猪、肉牛、大鹅等9大基地建设,不断提高优质化水平,每个县(市)区都要着力培育和发展1到2个真正有规模、有特色的优势产业。特别是要抓住实施“奶业振兴计划”以及伊利集团进入我市的良好机遇,举全市之力,全面提升我市乳业的产业化水平。充分发挥供销合作组织在推进农业产业化经营中的作用,引导乡镇企业重点发展农产品加工和储藏保鲜、运销业,使其通过体制创新、技术改造、布局优化和产业升级,提高市场竞争力。

大力发展订单农业,培育各种行业协会,加强农村各类经济合作组织、中介组织和经纪人队伍建设,逐步完善新型社会化服务体系。

强化基础,综合整治,深入推进农业的可持续发展。按照建设生态市的要求,加强农田水利工程建设,提高水资源控制和利用能力,重点搞好人畜饮水工程建设。进一步搞好小流域综合治理,防止水土流失。采取工程措施和生物措施,改善土壤结构,保护耕地资源,加大草原改良力度。搞好大型农机具和排灌设施更新,提高现代农业装备水平。

精心运作,务求实效,深入推进农村各项改革。巩固扩大农村税费改革和撤乡并村并校的成果,防止农民负担反弹。在稳定土地承包制度的前提下,建立健全土地流转机制,大力发展以农民股份制为重点的各类经济组织,推进土地规模化经营。同时,按照中央和省的统一部署,继续抓好粮食流通体制改革,加速粮食收储企业市场化和粮食经济产业化的进程,解决好农民卖粮难、企业销售难的问题。

(三)大力发展民营经济和第三产业,加速培育壮大新的经济增长点

继续实施大户牵动战略,促进民营经济扩规模上档次。真信真学真用浙闽经验,放手放胆放开加快发展。以产业政策为标准,在税收、信贷、融资、项目审批、土地使用、技改贴息、进出口贸易等方面与国有企业一视同仁。建立健全以资金融通、信用担保、管理咨询、信息服务、法律服务、市场开拓和人才培训等为主要内容的民营企业服务体系。以开通市和县(市)区领导与民营企业大户“办事直通车”为重点,建立和完善政府部门与民营企业联系、协调、服务制度。鼓励、支持民营企业以承包、租赁、联合、兼并、参股、控股、托管、收购等形式参与国有企业产权制度改革,促进民营企业快速扩张。积极引导和扶持个体私营经济向加工业转移,向旅游、信息、中介和社区服务等新兴产业延伸,向民营科技型企业方向迈进,向出口创汇型拓展。今年,全市个体工商税收力争增长8%。

立足启动扩大内需,加快发展第三产业。按照服务主体多元化、服务领域多向化、服务手段多样化的思路,加快发展信息、金融、会计、咨询、法律等现代服务业,改造提高商贸流通、交通运输、市政服务等传统服务业,突出发展医疗保健、家政服务、文化娱乐等社区服务业。同时,大力发展绿色生态、农业观光、金元文化、假日旅游等项目,加快安达牛城、绥化金龟山庄、兰西东林寺、望奎庙山、绥棱四海店、庆安柳河、北林民族风情观光等旅游区的景点建设,提高接待能力和质量,着力开发具有绥化特色的旅游商品,力争有更多的景点能够进入全省的旅游热线。培育扩大居民住房、汽车、旅游、通信、文化、体育等新的消费热点。

本着“谁投资、谁建设、谁受益”的原则,进一步强化市场体系建设。重点建设绿色食品、农副产品、地工产品等专业批发市场,逐步培育发展劳动力、人才、科技、信息、产权交易等生产要素市场。立足搞活市场流通,扶持优势企业占领市场,创立名牌产品拓展市场,寻找商机开辟外埠市场,建立稳定的销售体系,推进企业物流社会化、现代物流产业化。

(四)提高经济发展的外向度,构筑全方位对外开放的总体格局

举全市之力大搞招商引资,注重提高成功率、到位率和合同履约率。进一步改善招商引资的方式和手段,由以资源招商为主向以资源、项目招商为主转变,由以政府招商为主向以企业招商为主转变,由以大兵团招商为主向以专业队伍定向招商为主转变。坚持引资、引制、引智和引人才、引项目、引技术、引市场并重,引进内资与利用外资并举,把大上工业项目、绿色食品项目、高新技术项目、基础设施项目和第三产业项目作为招商引资的重点。今年,务求资金到位率达到50%以上,投产达效率超过历史最好水平,工业项目所占比重力争有较大提高。充分利用庆安举办“绿节”契机,“一处搭台、全市唱戏”,寻求广泛合作,扩大招商引联成果。

全面扩大对外经贸,努力在更大范围、更广领域参与国际经济技术合作和对外交流。发挥地缘优势,抓好对俄经贸合作,在开展农产品贸易和境外劳务及兴办企业上有新的突破。实行战略性引进、政策性引资和资源性招商有机结合,鼓励外商收购、兼并、参股、控股各种所有制企业。积极开拓以俄罗斯、东南亚、港澳台等国家和地区为重点的国际市场,大力开发粮油、畜禽产品、蔬菜瓜果、草柳编织、亚麻纺织等出口创汇产品。鼓励和支持轻工、建筑等有条件的企业走出国门,以加工贸易、劳务合作、资源开发、承包工程等形式,到境外创办、领办企业、扩大劳务输出。同时,进一步加强对外友好往来和文化、教育、科技等领域的合作与交流。

(五)全面推进科技进步和技术创新,努力提高经济运行质量

加强科技创新体系建设。广泛筹措资金,加大投入力度,积极创造条件,利用各种形式,加速构建新技术研究、开发、转化、推广体系。特别是要走好产学研结合的路子,借助外力尽快提高全市科技创新的整体水平。大力发展技术评估、技术市场等中介组织,加速科技成果市场化进程。有条件的企业要积极建立技术开发机构,把自主开发与引进消化吸收有机地结合起来,不断提高企业的自主技术创新能力。

加速用高新技术改造传统产业。重点抓好一批科技含量高、经济效益好、对市域经济发展有牵动能力的科教兴市标志性工程。工业要重点推广新技术、新材料、新工艺,改造食品、机械、纺织、建材等传统产业。今年,全市力争完成技改投资6.5亿元,完成技改项目55项。进一步强化农业技术创新和推广工作的力度,完善乡抓示范园、县抓示范区、市抓示范带的推广体系和工作机制,大力推广玉米、大豆、水稻标准化栽培技术。尤其要加强优质、高效农畜新品种的推广,良种覆盖率力争达到95%以上。

加快信息化建设进程。进一步完善全市网络结构,加大信息资源整合和开发利用力度,拓展网络应用范围,加快数字绥化建设步伐。积极推进电子政务,提高企业和农业的信息化水平,充分发挥各类专业网站的作用,推进经济社会各领域深度开发,广泛应用信息技术,把绥化推向加快发展的快车道。

加大人力资源开发力度。牢固树立“以人为本”的思想理念,在经济社会发展中改变重视物质资本投入轻视人力资本投入的倾向,把人力资源作为推进经济、社会、科技全面发展的第一资源来开发,不断提高回报率。增强目的性和有效性,制定和落实人力资源开发规划,建设学习型城市、学习型企业和学习型机关,大力提倡和推进“终生教育”,开展广泛系统的培训活动,尽快提高全体劳动者的科技文化素质和基本技能,为实现“六个规模推进”、加速科技创新、加快绥化发展提供足够的智力资源和人才保障。完善市场配置机制、利益驱动机制和柔性流动机制,广泛吸引各类人才,努力实现人力资源均衡开发利用。建立和完善多元化的人才发展和评价机制,加速培养懂经营、善管理、高技能和精于对外交往、善于攻坚克难、勇于开拓创新的人才,逐步形成一支适应市场经济发展和全方位对外开放需要的庞大的复合型人才队伍。

(六)加强财税金融工作,努力扩大发展建设资金的投入规模

突出重点,突破难点,提高财源建设总体效益。按照全省县级财源建设总体规划,精心组织好县乡财源建设。加大项目开发力度,争取有更多的优势项目进入全省财源建设的盘子。围绕“六个规模推进”,大力提高全市经济发展的质量和效益,千方百计巩固壮大现有财源,培育构筑新兴财源,充分挖掘潜在财源,把财政“蛋糕”做大。通过努力,争取再有一批乡镇进入财政收入水平超千万的行列,财政困难较大的县进一步提高自给能力,使全市的财源建设上升到一个新的档次。

深化改革,优化结构,提高财政工作管理水平。按照全市改革、发展、稳定的实际需要和建立公共财政的要求,深化以实行部门预算、国库集中支付、规范和扩大政府采购为重点的财政改革,加强预算管理,强化税收征管,调整和优化支出结构,严格监控预算外资金,坚持收支两条线,杜绝坐收坐支现象发生。适应农村税费改革的新形势,正确处理县乡之间的利益关系,理顺和完善乡镇财政体制。按照中央和省委关于艰苦奋斗、厉行节约的要求,把非生产性支出压缩到最低限度。

规范管理,防范风险,提高财政信贷资金运营质量。充分发挥金融部门作用,建设良好信用环境,建立资金支撑体系,搞好信贷结构调整,强化“三防一保”工作,加大对经济发展特别是企业的扶持力度。对农业开发项目、国债项目、世行贷款项目等各种财政和金融风险因素,严加监控,规范管理,促其按时达产达效,按时归还贷款本息,确保资金运营效益和质量。同时,加大审计监督力度,尤其要加强领导干部经济责任和国投、财政资金的审计,堵塞各种漏洞。

广辟渠道,广筹资金,提高建设发展资金投入能力。通过选好、选准项目,积极向上争取资金,吸引更多的外商投资;通过闲置资产的启动变现,以存量换增量,变死钱为活钱;通过改革投融资体制,落实促进民间投资的相关政策,放宽准入条件,广泛吸引和聚集更多的民间资金;通过强化城市经营,搞好土地置换,多渠道获取更多的建设资金。

(七)抓住国家扩大国债规模的机遇,积极争取项目投资

增发国债,实施积极的财政政策,是党和国家做出的扩大国内市场需求,确保经济稳定健康发展的一项重大决策。几年来,我市争取国债投资项目的总额已经达到12.2亿元,竣工和在建的项目共有41个。这些项目的建设,极大地改善了我市城乡的基础设施条件和总体经济结构,对经济社会发展产生了明显的拉动作用。今年国家继续实施积极的财政政策,国债增发规模将达到1400亿元,重点向重大基础设施建设、改善农村生产生活条件、促进企业技术进步和技术改造、中西部开发、生态环境建设、科教卫生和服务业发展等七个方面倾斜。我们要认真研究充分利用国家政策,及时掌握投资信息,千方百计做好项目争取和项目建设工作。

精心运作实施,加快在建工程进度。对已经开工建设尚未竣工投产的项目,认真分析原因,采取有效对策,加快工程建设进度。特别是由于地方匹配资金不到位,投入有缺口的,应采取多渠道融资的办法,确保资金及时到位,确保项目能够尽快地投产达效。对今年已经挤进国债“盘子”的项目,抓紧动工建设,力争早日竣工投入使用。特别是已经确定下来的12条县乡公路建设项目要抓紧组织实施。

加大工作力度,积极争取更多项目。在做好海伦大豆交易市场、庆安特色绿色市场、市现代农业示范园区以及人畜饮水、退耕还林、病险水库消险、城市供水、垃圾处理、高中扩招、农村中小学危房改造、农村扶贫等项目争取和建设的同时,力争把生态观光旅游、阁山水库、急救中心、社区服务中心、绿色食品监测检验中心、秸秆发电、集中供热等项目列入国家或省的计划。加大跑部进京、跑省进厅力度,争取国家和省给予更大的倾斜,获得更多的项目、资金和政策支持。

严格责任制度,确保项目投资效益。加强对国债项目的管理,坚持业主责任制、招投标制、监理制、合同制“四制”原则,明确政府、部门和项目建设单位的责任,精心组织施工,保证工程质量,强化资金监管,减少损失浪费,及时发现和解决工程建设中出现的各种问题,确保国债项目的质量标准和投资效益。

(八)加强社会主义精神文明建设,推进社会事业全面发展

切实加强思想道德和文化建设。坚持用邓小平理论和“三个代表”重要思想武装全市人民和广大干部,深入落实《公民道德建设实施纲要》,加强社会公德、职业道德和家庭美德教育,积极开展各种群众性精神文明创建活动,树立“爱国守法、明礼诚信、团结友善、勤俭自强、敬业奉献”的良好风尚。开展丰富多彩的先进、健康、有益的文化活动,扩大阵地,打造精品,规范市场,壮大队伍,把“黑土特色文化”建设和文化强市建设推向一个新的阶段。

深入落实教育优先发展的战略地位。优化教育结构和资源配置,完善管理体制,继续抓好“普九”教育,加大控辍力度,进一步扩大高中阶段教育,努力促进职业教育、成人教育。加大教育资金投入,抓好农村中小学校危房改造,改善办学条件,积极鼓励、大力支持社会力量办学。

全面提高城乡建设水平。以建设“绿色绥化”为目标,以区域中心城市建设为龙头,以小城镇建设为基础,以产业发育为支撑,以基础设施配套建设为重点,加速构筑城乡一体同步推进的新格局。高起点搞好城市规划,严格规划管理,发挥规划的龙头牵动作用。高标准搞好城市建设,规范建筑市场,严格审批程序,突出特色,形成风格,打造更多的精品工程和标志性工程,不断提高城市品位。高水平搞好城市管理,完善管理办法,加大依法治城力度,净化、美化、亮化城市环境,树立城市崭新形象。高效能搞好城市经营,探索市场化经营的路子,提高城市资产经营效益,解决建设资金投入不足的问题。特别是小城镇建设,更要积极探索市场化经营的路子,不断加大资金投入力度。同时,抓好绥兰路建设、绥望路路面铺装等重点工程。

认真实施可持续发展的基本国策。立足当前,着眼长远,正确处理人口、资源、环境与发展的关系。继续稳定低生育水平,提高出生人口素质。加快以“天保”工程为基础,以资源和生态保护为中心的生态示范市县建设,争取尽快达到国家验收标准。严格执行土地利用总体规划和年度计划,确保耕地总量动态平衡。

大力发展卫生、体育、广播电视等各项事业。优化卫生资源配置,加强医疗、药品市场管理和医德、医风建设,完善医疗服务体系,加快发展农村医疗卫生事业。进一步实施全民健身计划,提高竞技项目水平,增强国民身体素质。继续抓好广播电视基础设施建设,不断完善网络服务功能,充分发挥其在三个文明建设中的重要作用。同时,认真抓好民族宗教、外事侨务、防震减灾、残疾人和民兵预备役、双拥等各项工作。

(九)加速市本级经济和社会事业发展,增强带动和辐射功能

按照“工业领先、工贸齐兴,多元并举、开放开发,市区联动、共同发展”的总体思路,突出工作重点,转变领导方式,加大推进力度,在市本级经济总量扩张和整体功能完善上实现新突破。今年市本级规模以上工业增加值、利税同比分别增长9.5%和6%;财政总收入达到2.03亿元,一般预算收入6200万元,剔除变化因素,按可比口径同比分别增长7.4%和7.3%。

突出抓好“一区三园”建设。新城区建设,要坚持高标准、高起点搞好全面规划,从今年起逐步实施,分期建设。工业园区建设,力争完成3条横向道路、供排水、通信等“七通一平”工程。已经入区的绿色豆奶、三精制药项目争取尽快达产达效。加快马铃薯颗粒全粉、生物农药、营养餐、中药和饲料等项目的前期运作,争取再有3个—5个较大型的工业项目入区。农业示范园区建设,在完成总体规划和五个功能区规划立项的同时,积极创造条件,力争有1个—2个项目启动建设。民营科技园区要抓紧搞好规划,为企业入园做好准备。

加快改造提升现有企业。对北国酒业、水泥厂、外贸油脂厂等企业,通过联大靠强、活化资本、搞好技改和新产品开发,进一步扩大生产,增加效益;对绥化卷烟厂等中省直重点企业要加大扶持力度,努力实现满负荷生产;对八一厂等几家活力不强的市属企业,采取资产重组、租赁经营或分块突围的办法尽快搞活。

深入实施大商贸战略。以市场为取向,通过政策引导、资金扶持等办法,把果菜和小商品批发市场,建成辐射半径较大的专业批发市场;引导供销百货大楼、华辰商都等商贸企业逐步形成专营名店;把正大购物中心、中央商城办成特色突出的综合型知名商城;创造条件建立大型配送中心,实行上网交易,发展现代物流业;加快绿色食品、农用物资等专业市场的建设步伐。加强对市场建设的宏观调控,避免低水平重复建设和无序竞争。

强化区域中心城市建设。今年,市本级城市储备土地157.3公顷,采取招标拍卖和预出让供应土地11公顷,力争地产收益1500万元。重点抓好西二路、客运枢纽商服部分和红兴水库续建、货运枢纽二期工程和北二东路、中兴西路、西直路等主次干道维修改造和建设,做好垃圾处理、集中供热、秸秆发电等项目的前期工作,力争尽早开工建设。完成西二路两侧、前进小区、南城小区三个详细开发规划,并逐步实施推进。启动经济适用住房小区开发1处—2处。争取新辟建一处绿地,改造完善通信广场。在搞好环城林规划、城市街路、庭院绿化的基础上,重点搞好中直、西直、中兴东路主要街区门前绿化。树立“环境兴市”理念,以抓好城市综合整治为切入点,着重治理脏、乱、差,进一步整顿好交通秩序、社会公共秩序和市场交易秩序,继续治理烟尘和噪声污染,全面塑造城市发展的新形象。

大力推进市本级社会事业发展。重点提高教育、卫生、文化、广播电视、体育等社会事业发展水平。推进绥化师专建设综合性大学,做好肇东、海伦两个师范学校的升格工作,争取在市区新建一所高级中学,积极运作建设疾病防治控制中心,做好市第一医院急救中心建设和妇幼保健站设备改造工作,启动体育场馆建设。

(十)积极化解各种矛盾,全力做好稳定工作

坚持分级负责、归口办理的原则,深入做好群众来信来访工作。严格执行领导干部信访责任制和领导接待日制度,认真解决群众关心和反映的热点、难点问题,变群众上访为领导主动下访。加大依法治访力度,进一步规范信访工作秩序,提高依法处理信访问题的能力和水平。

采取综合有效措施,切实安排好城乡弱势群体和贫困群众的生活。按照十六大精神,切实抓好社保、下岗再就业、扶贫解困和老干部“两费”发放工作。组织全社会力量开展“一帮一、送温暖”活动,帮助下岗职工、低收入家庭、农村贫困户解决好生产生活的紧迫问题。

努力扩大社会就业,减轻政府和企业压力。认真落实下岗再就业的相关政策,搞好劳动力市场建设,规范用工行为,依法保护经营者和劳动者合法权益。积极发展劳动密集型产业和社区服务业,引导和鼓励下岗职工转变择业观念,提高自主择业能力,加速形成市场导向的就业机制。

切实搞好社会保障制度改革,扩大养老、失业、低保和医疗保险的覆盖面。完善社会保险征收体制,扩大社会保险覆盖面,确保养老金按时足额发放,积极稳妥推进下岗职工向失业保险并轨。今年,全市企业养老保险和失业保险覆盖面要达到100%,工伤、医疗、生育保险覆盖面要分别达到75%、60%和40%以上。

加强社会治安综合治理和安全生产工作,创建良好安定的生产生活环境。加强城市打防控一体化和农村“154”工程建设,全面实施“细胞工程”,依法严厉打击各类刑事犯罪活动和“法轮功”等邪教组织的非法活动,坚决扫除各种社会丑恶现象,保持良好的社会治安秩序。重点抓好防火、生产、交通等安全工作,加大综合整治力度,减少和杜绝各类安全事故的发生。

三、切实加强政府自身建设,不断提高领导能力和服务水平

完成今年的各项工作任务,必须认真实践“三个代表”重要思想,按照十六大提出的“四新”要求,努力建设勤政、廉洁、务实、高效的政府。

进一步解放思想,牢固树立开拓创新意识。注重引导教育广大干部对照先进找差距,自觉摆脱各种不适宜观念、做法和体制的束缚,树立与市场经济相适应的竞争意识、发展意识、效率意识、风险意识。坚持与时俱进,勇于开拓,做到在创新中求发展,在改革中攻坚克难,用创新思维、创新胆魄、创新方式,摆脱困难,排除干扰,开创各项工作的新局面。

进一步加强学习,切实增强决策指导能力。把学习作为做好新形势下政府工作的政治任务,坚持用邓小平理论、“三个代表”重要思想和十六大精神武装全体政府工作人员的头脑,增强贯彻执行党的路线、方针、政策的自觉性和坚定性。组织政府人员认真学习市场经济、科技、财税、金融、法律等知识,增强适应新形势、分析新情况、解决新问题的能力,提高按市场经济规律、客观规律和国际通行规则办事的能力。

进一步加强民主法制建设,坚持依法治市。自觉接受人民代表大会及其常委会的依法监督和人民政协的民主监督,认真办理人大代表的议案和政协委员的提案。密切政府同各民主党派、工商联、无党派人士以及人民团体的联系,认真听取建议和意见。主动接受群众监督、舆论监督和司法监督,尽快形成了解民情、反映民意、集中民智的良性监督机制,提高决策民主化、科学化水平。全面落实行政执法责任制和错案追究制,规范行政执法行为。

进一步转变政府职能,打造良好的经济发展环境。大力推行政务公开工作,建设“阳光”政府。严格实行限时办结制、失职追究制、服务承诺制、首问责任制、否定报备制、项目申报无偿代办制。继续开展“为经济建设服务、树行业新风”活动,改善服务环境。抓好治乱减负工作,强化法制环境建设。清理与市场经济不相适应的文件规定,用足用好用活国家和省出台的各项政策,抓好政策环境建设。着力解决政令不畅的问题,对干扰、障碍和破坏经济发展环境的部门和人员,一定要从严查处。

进一步转变工作作风,加大工作落实力度。大力改进领导方式方法,注重用市场机制、示范引导、典型引路指导各项工作。坚持求真务实,真抓实干,反对空谈,力戒形式主义。进一步精简会议、压缩文件,从文山会海和事务应酬中解脱出来,腾出更多时间和精力,抓落实,谋发展。大兴调查研究之风,深入基层,包扶企业,集中解决发展中的热点难点问题,把各项决策和部署不折不扣地落实到位。

进一步加强勤政廉政建设,树立政府良好形象。按照胡锦涛总书记“两个务必”的要求,恪守为民之责,善谋富民之策,做到情为民系、权为民用、事为民做,尽心竭力为人民群众办好事、谋利益。认真贯彻落实中央、省和市委制定的一系列廉政建设规定,切实加强政府机关领导干部廉洁自律工作,严肃查处违法违纪案件,坚决纠正部门和行业不正之风,标本兼治,从源头上预防和治理腐败。

各位代表,加快绥化各项事业发展,保持社会稳定,促进经济繁荣,是全市人民的期望和重托。我们要在市委的正确领导下,带领全市人民合力拼搏,奋发进取,为全面开创工作新局面,加快小康社会的建设步伐而努力奋斗!
